# A Multi-Dimensional Systematic Review of Minimally Invasive Bunion Surgery (MIBS)

**DOI:** 10.3390/jcm14082757

**Published:** 2025-04-17

**Authors:** Danielle Lonati, Ewan Kannegieter, Douglas McHugh

**Affiliations:** 1 Frank H. Netter MD School of Medicine, Department of Medical Sciences, Quinnipiac University, Hamden, CT 06518, USA; danielle.lonati@quinnipiac.edu (D.L.); e.kannegieter@nhs.net (E.K.); 2 Provide Health, Braintree Hospital, Braintree CM7 2AL, UK

**Keywords:** minimally invasive bunion surgery (MIBS), hallux valgus, bunionectomy, cost-effectiveness, patient satisfaction, rehabilitation protocols, surgical outcomes, radiographic correction, complication rates, quality of life

## Abstract

**Background/Objectives**: Hallux valgus, or a bunion, is a prevalent foot deformity associated with pain, limited mobility, and reduced quality of life. Surgical treatments include minimally invasive and traditional open techniques, but the optimal approach remains debated. This systematic review evaluates long-term outcomes, patient satisfaction, cost-effectiveness, the influence of patient-specific factors, rehabilitation protocols, and complication rates for these methods. **Methods**: A comprehensive search of PubMed, Medline, EMBASE, and Cochrane databases identified 22 studies published within the last 15 years, each with a minimum follow-up of 2 years. The systematic review adhered to PRISMA-ScR guidelines. Eligible studies reported on at least one of six key outcomes, and data were extracted on radiographic and clinical results, patient satisfaction, costs, rehabilitation timelines, and adverse events. **Results**: Minimally invasive bunion surgery (MIBS) showed faster recovery, higher patient satisfaction, and improved quality of life compared to open surgery. Radiographic outcomes, including hallux valgus and intermetatarsal angle correction, were durable, though outcomes were less consistent for severe deformities. MIBS was more cost-effective over time, owing to shorter operating times and faster recovery. Rehabilitation was accelerated, and wound complications were fewer with MIBS. However, open techniques remained preferable for severe deformities due to their reliability in complex corrections. **Conclusions**: MIBS offers substantial advantages for most patients undergoing bunion surgery, including faster recovery and fewer complications. However, open techniques may be better suited for severe deformities. Further research is needed to refine patient selection criteria and evaluate long-term outcomes in diverse populations.

## 1. Introduction

Hallux valgus, commonly known as a bunion, is a deformity characterized by medial deviation of the first metatarsal and lateral deviation with pronation of the big toe (hallux) [1]. This misalignment disrupts joint mechanics, leading to instability, pain, and a prominent bony protrusion at the first metatarsophalangeal (MTP) joint. Over time, progressive weakening of the medial supporting structures exacerbates joint misalignment and muscle imbalance, which can ultimately result in arthritis [1]. The most common etiologies include repetitive stress on the MTP joint and genetic predisposition, with factors such as weak connective tissue, a short Achilles tendon, tight calf muscles, and rheumatoid arthritis increasing the risk of bunion development [2]. Bunions affect approximately one in three individuals over the age of 65, occurring more frequently in women [2]. While some bunions are asymptomatic, many cause significant health issues, including pain, gait impairment, immobility, and an increased risk of falls [3]. Progressive deformity often leads to decreased quality of life, with alterations in gait affecting posture and causing discomfort in other areas of the body. Additionally, the cosmetic appearance of bunions and associated functional limitations may negatively impact social interactions and self-esteem [4].

Conservative treatments aim to alleviate symptoms but cannot correct the underlying deformity, leaving surgery as the definitive option [2]. Over 140 surgical techniques have been described, yet complications such as stiffness, recurrence, hardware irritation, avascular necrosis, and nerve entrapment remain prevalent [5,6].

In recent years, minimally invasive bunion surgery (MIBS) has gained traction in orthopedic and podiatric foot and ankle surgery [7,8,9]. MIBS offers several advantages, including reduced surgical trauma, better preservation of the local blood supply, faster recovery, and improved cosmetic outcomes (i.e., scarring) [9,10]. While earlier techniques were linked to high complication rates, advancements in methods and technology have significantly enhanced the reliability and safety of MIBS [8,9,10,11].

Traditional open techniques, on the other hand, involve larger incisions and more extensive dissection, which can lead to longer recovery periods and higher rates of postoperative stiffness, though they may provide more reliable correction for severe deformities due to greater structural visualization during the procedure.

Both approaches have demonstrated strengths and limitations depending on patient-specific factors, such as the severity of hallux valgus and comorbid conditions. The literature, however, lacks a comprehensive synthesis of the long-term outcomes, patient satisfaction, cost-effectiveness, and complications associated with MIBS in comparison to traditional techniques. This systematic review aims to address this gap by examining these key outcomes and offering insights into how MIBS performs relative to established surgical methods.

This systematic review aims to address this gap by analyzing the current evidence and exploring six specific questions regarding the outcomes of MIBS:

1. What are the long-term outcomes of MIBS in terms of correction stability, recurrence rates, and joint functionality?
2. How does MIBS for bunion correction affect patient satisfaction, quality of life, and return to daily activities and footwear compared to traditional surgery techniques?
3. From a healthcare economics perspective, how cost-effective is MIBS when considering surgical time, recovery period, complication rates, and long-term efficacy compared to conventional surgical approaches?
4. How do patient-specific factors such as age, sex, severity of deformity, and comorbidities influence the outcomes of MIBS?
5. What are the most effective rehabilitation and recovery protocols for MIBS, and how do they compare to those recommended after traditional bunion surgery?
6. What are the most common complications associated with MIBS, and what strategies can be implemented to minimize these risks?

The goal is to identify factors contributing to optimal results for both patients and surgical teams, ultimately informing clinical decision-making and improving patient care.

## 2. Materials and Methods

### 2.1. Search Strategies

A systematic and comprehensive search was conducted using the PubMed, Medline, EMBASE, and Cochrane databases to identify relevant studies comparing MIBS and traditional open surgery. The search targeted six specific aims: (1) long-term outcomes and stability, (2) patient satisfaction and quality of life, (3) cost-effectiveness, (4) the influence of patient-specific factors, (5) rehabilitation and recovery, and (6) complications.

The search strategy incorporated the terms (“minimally invasive surgery” OR MIS) AND (bunionectomy OR bunion surgery) combined with aim-specific terms. These included the following:

- Aim 1: (long-term outcomes OR stability OR correction stability);
- Aim 2: (patient satisfaction OR quality of life);
- Aim 3: (cost-effectiveness OR economic evaluation);
- Aim 4: (patient-specific factors OR demographics OR comorbidities) AND (surgical success OR outcomes);
- Aim 5: (rehabilitation OR recovery protocols OR postoperative care);
- Aim 6: (complications OR adverse events OR management).

Duplicates were removed, and articles were screened by title and abstract for relevance. Full-text articles meeting inclusion criteria were subsequently reviewed for data extraction. Studies included in this review were required to have a minimum follow-up period of two years and address at least one of the six aims.

**Figure 1:**
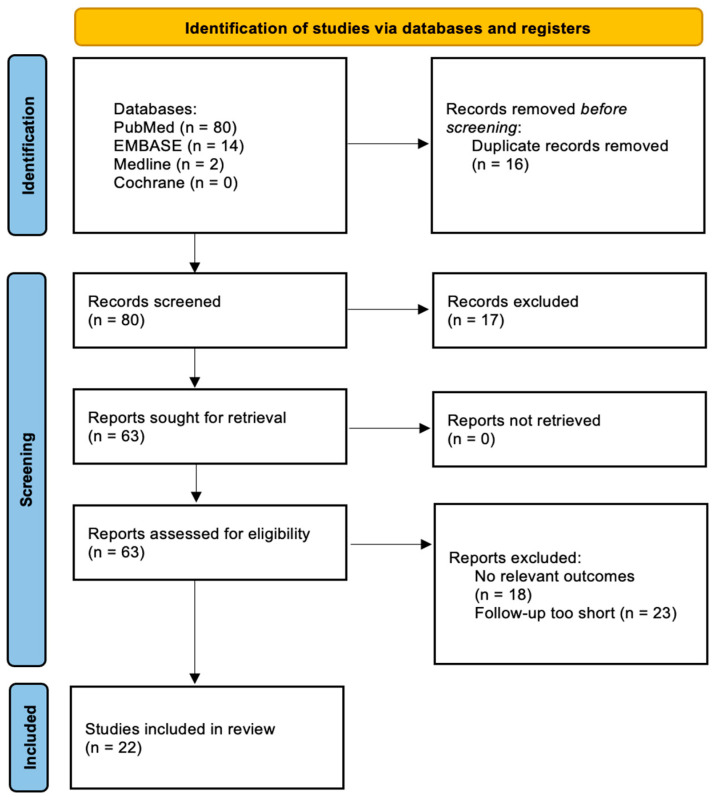
PRISMA flow diagram depicting the process of literature selection and screening.

The entire systematic review process followed the PRISMA-ScR checklist to ensure methodological rigor and adherence to systematic review standards [12]. A PRISMA flow diagram (Figure 1) provides a visual representation of the screening and selection process. This review was not registered.

### 2.2. Inclusion and Exclusion Criteria

Studies were included if they met the following criteria: peer-reviewed research articles or clinical trial results focusing on MIBS, reporting outcomes relevant to one or more of the six specified aims, a minimum follow-up duration of two years, and publication in English within the past 15 years.

Exclusion criteria encompassed opinion pieces, editorials, and non-peer-reviewed articles; studies that did not address outcomes related to the six aims; studies with follow-up periods shorter than two years; non-English publications; and studies published more than 15 years ago.

### 2.3. Data Extraction and Quality Assessment

Authors D.L. and D. M. categorized the included articles according to *The Journal of Bone & Joint Surgery*’s published level-of-evidence criteria [13]. This hierarchical classification of the strength of evidence corresponds to the quality and reliability of the study design, as outlined in evidence-based medicine frameworks:

- Level I: High-quality RCTs with strong methodology and controls, or high-quality evidence from systematic reviews or meta-analyses of RCTs.
- Level II: Lesser-quality RCTs, prospective cohort studies with a comparison group, or systematic reviews of Level II studies.
- Level III: Retrospective cohort studies with comparison groups, case–control studies, or systematic reviews of Level III studies.
- Level IV: Case series or retrospective studies without comparison or control groups, often descriptive in nature.
- Level V: Expert opinions, narrative reviews, or theoretical/basic science research without clinical validation.

D.L. conducted the data extraction. Extracted data included the following: author, year of publication, study title, study design, length of follow-up, and outcomes pertinent to the six aims of this review—long-term outcomes and stability, patient satisfaction and quality of life, cost-effectiveness, influence of patient-specific factors, rehabilitation and recovery, and complications of MIBS versus open surgery.

The included studies employed diverse methodologies, including systematic reviews, meta-analyses, randomized controlled trials, retrospective studies, and case series, with evidence levels ranging from I to V [13].

## 3. Results

### 3.1. Synopsis of Reviewed Articles

This systematic review analyzed 22 articles published between 2009 and 2024, examining various aspects of MIBS for hallux valgus (Table 1). Key outcomes evaluated included functional and radiographic success, learning curves, complication rates, and comparative effectiveness between MIBS and traditional open surgical techniques.

Collectively, the findings provide a comprehensive overview of the safety, efficacy, and advancements in MIBS for hallux valgus. The studies highlight the benefits of MIBS, including faster recovery, fewer complications, and improved patient satisfaction in comparison to open techniques, while also addressing the challenges associated with its adoption, such as the learning curve and variability in outcomes for severe deformities.

### 3.2. Aim 1—Long-Term Outcomes: Correction Stability, Recurrence Rates, and Joint Functionality

**Figure 2:**
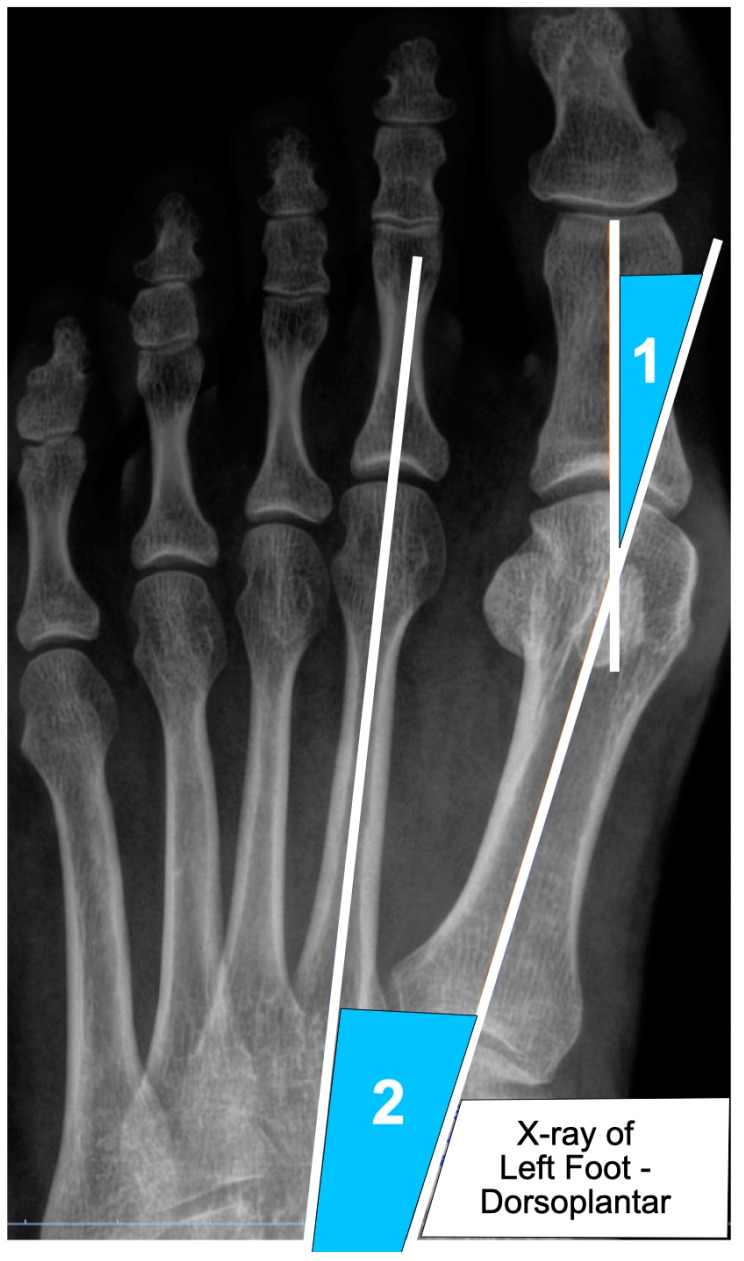
Dorsoplantar X-ray radiograph of the (1) hallux valgus angle (HVA) and (2) intermetatarsal angle (IMA); the HVA is measured as the angle formed between the longitudinal axis of the first metatarsal and the proximal phalanx of the hallux; the IMA is measured as the angle formed between the longitudinal axes of the first and second metatarsal bones.

The objective of Aim 1 was to evaluate the long-term outcomes of MIBS, focusing on correction stability, recurrence rates, and joint functionality (Table 2). Most studies included in our review utilized consistent radiographic assessments, specifically the hallux valgus angle (HVA) and intermetatarsal angle (IMA), as benchmarks for surgical success (Figure 2).

Overall, MIBS for moderate to severe hallux valgus demonstrated durable outcomes, significantly reducing both the HVA and IMA with low recurrence rates at an average follow-up of 97 months [15]. For instance, Lewis et al. reported that MIBS decreased the HVA from 27.2° to 7.2° (*p* < 0.001) and IMA from 12.0° to 6.0° (*p* < 0.001) postoperatively, with a recurrence rate of just 7.7% at a mean follow-up of 66.8 months [25]. This recurrence rate was comparable to previously published rates for open surgical techniques.

When comparing MIBS to open surgery, the results were mixed. One study with a 14-year follow-up showed that MIBS achieved significantly greater IMA correction compared to open surgery (−5.2° vs. −3.6°, *p* = 0.007). However, no substantial differences in long-term functionality or recurrence were observed between the two approaches [19]. Both techniques exhibited a similar tendency for deterioration and relatively high recurrence rates over time.

In contrast, a meta-analysis involving thousands of patients found that the MIBS group had significantly higher rates of excellent or good radiographic outcomes compared to open surgery [27]. While this suggests that MIBS may be preferable for hallux valgus correction, further stratification by deformity severity revealed that open techniques may yield better outcomes in severe cases.

### 3.3. Aim 2—Patient Satisfaction and Quality of Life

Aim 2 explored differences between MIBS and traditional open surgical techniques in terms of patient satisfaction, quality of life, and return to daily activities (Table 3). The primary measures of clinical outcomes used in these studies were the American Orthopedic Foot and Ankle Society (AOFAS) score and the Manchester Oxford Foot Questionnaire (MOXFQ), alongside various other patient-reported outcome measures (PROMs). The AOFAS score ranges from 0 to 100, with higher scores indicating better function and less pain, whereas the MOXFQ score also ranges from 0 to 100, but higher scores signify greater disability. These metrics allowed studies to assess patient pain, foot function, and the impact of surgery on daily life at various time points before and after surgical intervention.

In general, MIBS was found to significantly improve patients’ pain levels and quality of life. For example, one study reported a mean AOFAS score improvement from 57.9 preoperatively to 90.5 postoperatively, with sustained high scores over time [23,25]. Similarly, MIBS demonstrated substantial improvements in all domains of the MOXFQ score, reducing the mean MOXFQ score by 34.5 points at two years post-surgery [24]. These improvements were typically most pronounced at six months postoperatively and remained stable over two years [19,24].

However, long-term follow-up data from a randomized controlled trial by Jeuken et al. showed no significant differences in AOFAS, MOXFQ, or pain scores between MIBS and open surgery [27]. This finding contrasts with results from other meta-analyses and reviews, which consistently reported significantly higher AOFAS scores and lower early postoperative pain scores for MIBS compared to open surgery [14,21,27]. Patient satisfaction was also notably higher in the MIBS groups, driven by shorter scar lengths, improved cosmesis, reduced stiffness, and the ability to bear weight immediately postoperatively [21,23,24,27].

Overall, studies that directly compared MIBS to open techniques consistently found higher patient satisfaction and improved quality of life among patients undergoing minimally invasive surgery for bunion correction.

### 3.4. Aim 3—Cost-Effectiveness

Aim 3 examined the cost-effectiveness of MIBS by evaluating surgical time, recovery period, complication rates, and long-term efficacy compared to conventional open surgical approaches (Table 4). A critical factor influencing these statistics is the learning curve associated with adopting a new surgical technique. As MIBS has been reintroduced more recently, surgeons must undergo a learning phase to achieve technical proficiency. During this phase, operating room (OR) time and fluoroscopy use are significantly higher [25,30,34]. Studies suggest that proficiency is typically attained after 30–38 cases, at which point both surgical duration and radiation exposure decrease substantially [29,34]. This highlights the importance of surgeon experience in optimizing MIBS efficiency. However, despite the increased OR time during the learning phase, studies have found no associated rise in complication rates or compromise in patient outcomes [30].

Once the learning phase is surpassed, MIBS demonstrates cost-effectiveness compared to traditional open techniques. Lu et al.’s meta-analysis reported shorter recovery times in the MIBS group, along with several advantages of reduced surgical exposure and tissue dissection, such as shorter surgical duration, less postoperative pain, and quicker rehabilitation [27]. These findings are consistent with Maffulli et al.’s systematic review, which concluded that MIBS offers comparable efficacy to open surgery while incurring fewer costs and shorter operative times [28]. Additionally, a more recent meta-analysis confirmed equivalent complication rates between MIBS and open surgery but highlighted shorter surgical duration and hospital stays for MIBS patients [14].

Overall, the evidence suggests that while the learning phase of MIBS requires a higher initial investment in terms of time, the technique is both efficient and cost-effective once proficiency is achieved, offering faster recovery and reduced healthcare utilization.

### 3.5. Aim 4—Role of Patient-Specific Factors

Aim 4 investigated how patient-specific factors—including age, sex, severity of deformity, and comorbidities—affect the outcomes of MIBS (Table 5). Although research on this topic remains limited, several studies provide insights into the influence of these factors.

The most consistently observed trend was the relationship between deformity severity and surgical outcomes. Patients with more severe hallux valgus deformities had significantly lower postoperative American Orthopedic Foot and Ankle Society (AOFAS) scores compared to those with mild to moderate deformities. However, a loss of correction was observed for larger deformities in both MIBS and open surgical techniques [15,27]. This suggests that for severe deformities, an open approach or consideration of a first metatarso-cuneiform fusion—either open or minimally invasive—may be necessary for optimal correction.

Regarding other factors, age showed no correlation with radiological outcomes in MIBS, but sex demonstrated some trends. Male patients achieved greater correction of the hallux valgus angle (HVA) postoperatively (*p* = 0.041), while female patients experienced more effective and sustained correction over time (*p* = 0.047) [15]. Obesity did not correlate with postoperative AOFAS scores, radiological correction, or complication rates [17,33]. However, diabetic patients exhibited a significantly higher infection rate compared to non-diabetic patients, regardless of weight (9% vs. 2%, *p* = 0.03) [33].

Notably, the shorter surgical duration and reduced hospital stays associated with MIBS make it a promising option for medically compromised patients [28].

### 3.6. Aim 5—Rehabilitation and Recovery Protocols

Aim 5 evaluated the most effective rehabilitation and recovery protocols following MIBS and compared them to those used after traditional open surgery (Table 6). Although detailed recovery protocols for each technique are not extensively documented, significant differences in postoperative recommendations are evident.

For open surgical correction of hallux valgus, patients are typically advised to avoid weight-bearing activities for at least two weeks, often longer. In contrast, MIBS allows for immediate weight-bearing and limited walking postoperatively, often facilitated by the use of a specialized postoperative shoe [16,18,35]. Even with a complete reliance on MIBS, patients can safely bear weight soon after surgery [18].

The reduced recovery and rehabilitation times associated with MIBS are attributed to the less invasive nature of the procedure, which involves minimal surgical exposure and soft tissue dissection compared to open techniques. This enables patients to resume daily activities more quickly and with greater ease.

### 3.7. Aim 6—Incidence and Management of Complications

The objective of Aim 6 was to identify the most common complications associated with MIBS and explore strategies to minimize these risks (Table 7). Reported complications vary across studies, even for similar techniques. Brogan et al. reported a 7.8% overall rate of serious complications requiring a return to the operating room, with issues such as osteotomy displacement, delayed union, prominent screws, and infection [16].

The most frequently reported complications, identified in multiple studies, include joint stiffness, hallux valgus recurrence, first metatarsal shortening, material intolerance, osteoarthritic changes, infection, and transfer metatarsalgia [21,25,28,30,31]. Of these, joint stiffness and recurrence are the most common [31]. Several studies agree that these complications are often linked to improper execution of surgical techniques rather than inherent flaws in the procedures themselves. This is supported by a notable reduction in complication rates as surgeons progress beyond the learning phase [28,30].

Comparing MIBS to open procedures, the incidence of complications is generally similar [27]. Studies have found no statistically significant differences between the techniques in rates of revision surgery, recurrence, screw removal, paresthesia, or stiffness [16]. However, one study reported that in an overall complication rate of 19%, 70% of the complications occurred in patients who underwent open procedures (*p* = 0.042) [35].

These findings suggest that MIBS has a comparable safety profile to open surgery, with proper surgical training and experience being key to reducing complications.

## 4. Discussion

This systematic review highlights the efficacy of MIBS across multiple dimensions, with particularly strong outcomes in patient satisfaction, expedited recovery, and cost-effectiveness. However, traditional open techniques remain a viable option, especially for severe hallux valgus cases where they may offer superior structural correction. Across the six aims, MIBS consistently outperformed or was comparable to open techniques, demonstrating its versatility and potential as a first-line option for most patients.

A key limitation of the current evidence base, however, is the small number of studies that provide direct, high-quality comparisons between MIBS and traditional open techniques, specifically those of Brogan et al. (2016) [16], Jeuken et al. (2016) [19], Ji et al. (2022) [21], Lu et al. (2020) [27], and Cardoso et al. (2021) [35]. Of the studies included in this review, only a few directly addressed comparative outcomes, and these studies often varied in sample size, follow-up duration, and surgical technique. This heterogeneity complicates direct comparisons and impacts the generalizability of findings. Consequently, the conclusions we draw regarding the relative advantages of MIBS must be interpreted within the context of these limitations. Further research—particularly well-designed randomized controlled trials that directly compare MIBS and open approaches—is necessary to strengthen the evidence and provide more definitive guidance for clinical decision-making.

### 4.1. Long-Term Outcomes and Stability of Correction

The long-term outcomes of MIBS for hallux valgus are promising, particularly for mild to moderate deformities. Studies consistently report that MIBS effectively reduces the HVA and IMA with low recurrence rates, comparable to or exceeding the results achieved with open techniques [15,25,27]. However, the severity of the deformity influences outcomes, with open surgery potentially providing more durable corrections in severe hallux valgus cases [27]. Despite these variations, the overall stability achieved with MIBS in appropriate cases supports its growing use as a viable alternative to open approaches.

### 4.2. Patient Satisfaction and Quality of Life

Patient satisfaction and quality of life are critical measures of success in bunion surgery, and our findings highlight the significant benefits of MIBS in these areas. Across multiple studies, MIBS demonstrated faster postoperative recovery times, reduced pain, and shorter hospital stays [14,23,27]. These improvements were reflected in higher AOFAS scores and other patient-reported outcomes, with sustained gains in function and pain relief over time [22,24,25]. The reduced invasiveness of MIBS also led to fewer complications such as wound issues and stiffness, allowing for quicker rehabilitation and return to daily activities. These results underscore the growing preference for MIBS among patients and clinicians seeking optimal long-term outcomes with minimal recovery burden [27].

### 4.3. Cost-Effectiveness

Cost-effectiveness is a crucial factor in the growing adoption of MIBS. While the learning curve for MIBS initially results in longer operating times and increased fluoroscopy use, these challenges diminish as surgeons gain experience. Once proficiency is achieved, MIBS offers shorter surgical durations, reduced hospital stays, and faster recoveries compared to open techniques [14,23,27]. These factors translate to significant cost savings by minimizing postoperative care requirements and enabling earlier returns to daily activities. Additionally, the less invasive nature of MIBS reduces postoperative complications, further enhancing its cost-effectiveness.

### 4.4. Role of Patient-Specific Factors

Patient-specific factors such as deformity severity, sex, and comorbidities play a critical role in the success of MIBS. While MIBS generally yields favorable outcomes, its efficacy may be limited for patients with severe hallux valgus, where open techniques might be more suitable [27]. Sex-specific trends also highlight the importance of individualized treatment planning, with female patients often achieving more sustained corrections [25]. Comorbidities like diabetes increase the risk of complications, emphasizing the need for careful patient selection and tailored postoperative management to optimize outcomes [33].

### 4.5. Rehabilitation and Recovery Protocols

One of the most significant advantages of MIBS is its streamlined rehabilitation process. Unlike open surgery, where weight-bearing is typically delayed for weeks, MIBS patients can begin weight-bearing immediately after surgery [16,18,32]. This early mobilization accelerates recovery, reduces complications such as joint stiffness, and allows for a quicker return to daily activities. The reduced soft tissue dissection in MIBS contributes to faster healing and less postoperative discomfort, offering a more patient-friendly recovery experience.

### 4.6. Complications and Safety Profile

The complication rates associated with MIBS are comparable to those of open surgery, with no significant differences in the rates of recurrence, revision, or stiffness [27,35]. However, some studies suggest a lower incidence of wound complications and nonunion in MIBS [16]. The learning curve associated with MIBS is a critical factor in minimizing complications, as experienced surgeons achieve significantly lower rates of adverse events. While both techniques carry inherent risks, the less invasive nature of MIBS makes it a potentially safer alternative for appropriate patients.

### 4.7. Strengths, Limitations, and Future Directions

This systematic review’s inclusion of studies with extensive follow-up periods provides robust insights into the long-term outcomes of MIBS. However, a key limitation of our findings is the heterogeneity of both open and MIBS procedures. Techniques such as Chevron, Scarf, and Lapidus differ significantly in terms of surgical approach, recovery timelines, and postoperative care. This variation complicates the direct comparison of outcomes across studies and highlights a need for greater consistency in research methodologies. Future studies should aim to standardize procedures and outcome reporting to enable more reliable comparisons and evidence-based decision-making for surgical approaches.

The rapid evolution of MIBS techniques also suggests that newer studies may yield different results. Future research should focus on randomized controlled trials comparing MIBS and open techniques in patients with severe hallux valgus, as well as long-term studies assessing quality of life and functional outcomes. Investigating the impact of patient-specific factors, such as comorbidities and demographics, could further refine surgical decision-making.

## 5. Conclusions

This systematic review underscores the growing evidence supporting MIBS as a highly effective option for bunion correction, particularly in terms of patient satisfaction, recovery, and long-term correction stability. Faster recovery times, reduced pain, and fewer complications make MIBS an attractive option for both patients and clinicians. However, procedural heterogeneity remains a challenge in comparing outcomes across different MIBS techniques. Future research should prioritize standardized protocols to enhance the comparability and reliability of results, thereby refining clinical recommendations for optimal technique selection.

## Figures and Tables

**Table 1:** Summary of key data from the 22 articles included in this review, ordered alphabetically by first author’s last name.

Ref.	Authors	Year	Level of Evidence	Study Type	Journal	Article Title
[14]	Baumann et al.	2023	I	Systematic Review	*Foot and Ankle Surgery*	Learning curve associated with minimally invasive surgery for hallux valgus
[15]	Biz et al.	2021	IV	Case Series	*Foot & Ankle International*	Functional and Radiographic Outcomes of MIIND for Moderate to Severe Hallux Valgus
[16]	Brogan et al.	2016	III	Retrospective Cohort Study	*Foot & Ankle International*	Minimally Invasive and Open Distal Chevron Osteotomy for Mild to Moderate Hallux Valgus
[17]	Carlucci et al.	2021	III	Retrospective Cohort Study	*Foot and Ankle Surgery*	Is obesity a risk factor in percutaneous hallux valgus surgery?
[18]	Del Vecchio et al.	2020	I	Systematic Review	*Foot and Ankle Clinics*	Evolution of Minimally Invasive Surgery in Hallux Valgus
[19]	Jeuken et al.	2016	II	Randomized Controlled Trial	*Foot & Ankle International*	Long-term Follow-up Comparing Scarf to Chevron Osteotomy in Hallux Valgus Correction
[20]	Jeyaseelan et al.	2020	III	Systematic Review	*Foot and Ankle Clinics*	Minimally Invasive Hallux Valgus Surgery—A Systematic Review
[21]	Ji et al.	2022	II	Systematic Review and Meta-Analysis	*Frontiers in Surgery*	Minimally Invasive vs. Open Surgery for Hallux Valgus: A Meta-Analysis
[22]	Lausé et al.	2023	V	Narrative Review	*Journal of the American Academy of Orthopaedic Surgeons*	Minimally Invasive Foot and Ankle Surgery: A Primer for Orthopaedic Surgeons
[23]	Lewis et al.	2022	IV	Case Series	*Foot and Ankle Surgery*	Minimally invasive surgery for severe hallux valgus in 106 feet
[24]	Lewis et al.	2022	IV	Case Series	*Foot and Ankle Surgery*	Time to maximum clinical improvement following minimally invasive chevron and Akin osteotomies (MICA) in hallux valgus surgery
[25]	Lewis et al.	2023	III	Retrospective Cohort	*Foot & Ankle International*	Five-Year Follow-up of Third-Generation Percutaneous Chevron and Akin Osteotomies (PECA) for Hallux Valgus
[26]	Lewis et al.	2023	III	Retrospective Cohort	*The Journal of Foot and Ankle Surgery*	The Learning Curve of Third-Generation Percutaneous Chevron and Akin Osteotomy (PECA) for Hallux Valgus
[27]	Lu et al.	2020	II	Systematic Review and Meta-Analysis	*The Journal of Foot and Ankle Surgery*	Comparison of Minimally Invasive and Traditionally Open Surgeries in Correction of Hallux Valgus: A Meta-Analysis
[28]	Maffulli et al.	2011	III	Systematic Review	*British Medical Bulletin*	Hallux valgus: effectiveness and safety of minimally invasive surgery. A systematic review
[29]	Mazzotti et al.	2024	IV	Retrospective Observational Case Series	*The Journal of Foot and Ankle Surgery*	Combined Distal Metatarsal and Akin Osteotomies for Concomitant Metatarsophalangeal and Interphalangeal Hallux Valgus: Clinical and Radiological Outcomes
[30]	Merc et al.	2023	IV	Retrospective Observational Case Series	*BMC Musculoskeletal Disorders*	Learning curve in relation to radiation exposure, procedure duration and complications rate for Minimally Invasive Chevron Akin (MICA) osteotomy
[31]	Miranda et al.	2021	III	Systematic Review	*The Journal of Foot and Ankle Surgery*	Complications on Percutaneous Hallux Valgus Surgery: A Systematic Review
[32]	Oliva et al.	2009	V	Narrative Review	*Orthopedic Clinics of North America*	Minimally Invasive Hallux Valgus Correction
[33]	Stewart et al.	2016	III	Retrospective Cohort Study	*American Orthopaedic Foot & Ankle Society*	Effect of Obesity on Outcomes of Forefoot Surgery
[34]	Toepfer et al.	2022	IV	Retrospective Observational Case Series	*Foot and Ankle Surgery*	The percutaneous learning curve of 3rd generation minimally-invasive Chevron and Akin osteotomy (MICA)
[35]	Vieira Cardoso et al.	2022	III	Retrospective Cohort Study	*Foot & Ankle International*	Cohort Comparison of Radiographic Correction and Complications Between Minimal Invasive and Open Lapidus Procedures for Hallux Valgus

**Table 2:** Summary of key data from the 5 articles included for Aim 1 of this systematic review.

Ref.	Authors/Year	Level of Evidence	Follow-Up (Mean Months)	HVA at Last Follow-Up (Mean ± SD)	IMA at Last Follow-Up (Mean ± SD)	Recurrence Rate (%)
[15]	Biz et al. (2021)	IV	97	12.2 ± 8.2	6.4 ± 3.3	6%
[19]	Jeuken et al. (2016)	II	165.6	19.8	8.3	73%
[26]	Lewis et al. (2023)	III	66.8	7.8 ± 5.1	6.0 ± 2.6	7.7%
[27]	Lu et al. (2020)	II	-	Excellent–Good radiographic angular results: MIBS > Open OR = 6.28; CI 3.20 to 12.32 (*p* < 0.01)		-

**Table 3:** Summary of patient-reported outcome measures (PROMS): patient satisfaction, American Orthopedic Foot and Ankle Society (AOFAS), and Manchester Oxford Foot Questionnaire (MOXFQ) data from the 8 articles included for Aim 2 of this systematic review. RR = risk ratio. SMD = standardized mean difference. CI = confidence interval.

Ref.	Authors/Year	Level of Evidence	Follow-Up (Mean Months)	Patient Satisfaction (%)	AOFAS (0–100)	MOXFQ (0–100)
[15]	Biz et al. (2021)	IV	97	87%	90.5	-
[19]	Jeuken et al. (2016)	II	165.6	64%	MIBS = Open (*p* = 0.540)	MIBS = Open (*p* = 0.634)
[20]	Jeyaseelan et al. (2020)	III	30.2	MIBS > Open 87–94%	Improved from 18.1 to 66.1	-
[21]	Ji et al. (2022)	II	-	MIBS > Open RR = 1.15; CI 1.05 to 1.27 (*p* = 0.002)	MIBS > Open SMD = 0.45; CI 0.03–0.87 (*p* = 0.04)	-
[23]	Lewis et al. (2022)	IV	24	-	-	6.3
[24]	Lewis et al. (2022)	IV	24	-	-	6.7
[25]	Lewis et al. (2023)	III	66.8	77.4%	-	10.1
[27]	Lu et al. (2020)	II	-	52.6%	-	-

**Table 4:** Summary of perioperative and postoperative outcome measures from the 9 articles included for Aim 3 of this systematic review. OR = odds ratio. SMD = standardized mean difference. CI = confidence interval.

Ref.	Authors/Year	Level of Evidence	Follow-Up (Mean Months)	Surgical Time (Mean Minutes)	Recovery Period (Days in Hospital)	Complication Rates (%)
[14]	Baumann et al. (2023)	I	-	58.7	-	3.3–17%
[20]	Jeyaseelan et al. (2020)	III	-	-	-	4–19%
[21]	Ji et al. (2022)	II	-	MIBS < Open SMD = −2.81; CI −3.55 to −2.07 (*p* < 0.001)	-	MIBS = Open RR = 1.09; CI 0.77 to 1.56) (*p* = 0.02)
[25]	Lewis et al. (2023)	III	-	62.6	-	8.6%
[27]	Lu et al. (2020)	II	-	-	MIBS = Open SMD = −3.09; CI −7.98 to 1.80 (*p* = 0.22)	MIBS = Open OR = 0.67; CI 0.24 to 1.91 (*p* = 0.45)
[28]	Maffulli et al. (2011)	III	25.9	-	1.3	12.2%
[29]	Mazzotti et al. (2024)	IV	27.1	16.5	-	7.1%
[30]	Merc et al. (2023)	IV	-	47.0	-	22%
[34]	Toepfer et al. (2022)	IV	-	46.8	-	0.1%

**Table 5:** Summary of various effect modifiers from the 5 articles included for Aim 4 of this systematic review.

Ref.	Authors/Year	Level of Evidence	Follow-Up (Mean Months)	Effect of Severity of Deformity	Effect of Patient Sex	Effect of Diabetes Status
[15]	Biz et al. (2021)	IV	97	Milder severity was associated with better surgical outcomes and patient satisfaction.	Female sex was observed to be associated with effective correction of the HVA after surgery and its persistence over time.	-
[17]	Carlucci et al. (2021)	III	29	-	-	-
[27]	Lu et al. (2020)	II	-	Greater severity of deformity leads to poor MIBS outcomes.	-	-
[28]	Maffulli et al. (2011)	III	25.9	-	-	-
[33]	Stewart et al. (2016)	III	-	-	-	Diabetic patients had significantly higher rates of infection.

**Table 6:** Summary of postoperative recovery measures from the 4 articles included for Aim 5 of this systematic review.

Ref.	Authors/Year	Level of Evidence	Follow-Up (Mean Months)	Time Until Weight-Bearing	Time Until Physical Therapy
[16]	Brogan et al. (2016)	III	24–58	Heel weight-bearing: immediately Full weight-bearing: 4 weeks	-
[18]	Del Vecchio et al. (2020)	I	-	With postoperative rigid shoe: immediately	-
[32]	Oliva et al. (2009)	V	-	With postoperative rigid shoe: immediately	Immediately
[35]	Vieira Cardoso et al. (2022)	III	29	Heel weight-bearing: immediately Full weight-bearing: 2 weeks	Immediately

**Table 7:** Summary of postoperative outcomes measures from the 8 articles included for Aim 6 of this systematic review.

Ref.	Authors/Year	Level of Evidence	Follow-Up (Mean Months)	Complication Rates (%)	Recurrence Rates (%)	Joint Stiffness
[15]	Biz et al. (2021)	IV	97	15%	6%	-
[16]	Brogan et al. (2016)	III	24–58	MIBS = Open (*p* > 0.5)	MIBS = Open (*p* = 1.00)	MIBS = Open (*p* = 0.67)
[22]	Lausé et al. (2023)	V	-	7.8%	-	-
[27]	Lu et al. (2020)	II	-	MIBS = Open (*p* = 0.45)	-	-
[28]	Maffulli et al. (2011)	III	25.9	12.2%	-	-
[30]	Merc et al. (2023)	IV	-	22%	2%	1%
[31]	Miranda et al. (2021)	III	42.3	23.0%	15.2%	18.5%
[35]	Vieira Cardoso et al. (2022)	III	29	10.6%	4.3%	-

## Data Availability

The data presented in this study are available on request from the corresponding author.

## Abbreviations

The following abbreviations are used in this manuscript:

AOFAS	American Orthopedic Foot and Ankle Society
CI	Confidence Interval
HVA	Hallux Valgus Angle
IMA	Intermetatarsal Angle
MIBS	Minimally Invasive Bunion Surgery
MOXFQ	Manchester Oxford Foot Questionnaire
MTP	Metatarsophalangeal
OR	Odds Ratio
PRISMA	Preferred Reporting Items for Systematic Reviews and Meta-Analyses
RCT	Randomized Clinical Trial
RR	Risk Ratio
SMD	Standardized Mean Difference
